# Perception of Prosodic Modulations of Linguistic and Paralinguistic Origin: Evidence From Early Auditory Event-Related Potentials

**DOI:** 10.3389/fnins.2021.797487

**Published:** 2021-12-23

**Authors:** Hatice Zora, Valéria Csépe

**Affiliations:** ^1^Max Planck Institute for Psycholinguistics, Nijmegen, Netherlands; ^2^Brain Imaging Centre, Research Centre for Natural Sciences, Budapest, Hungary

**Keywords:** linguistic prosody, affective prosody, pitch accent, EEG, MMN, P3a, LPC

## Abstract

How listeners handle prosodic cues of linguistic and paralinguistic origin is a central question for spoken communication. In the present EEG study, we addressed this question by examining neural responses to variations in pitch accent (linguistic) and affective (paralinguistic) prosody in Swedish words, using a passive auditory oddball paradigm. The results indicated that changes in pitch accent and affective prosody elicited mismatch negativity (MMN) responses at around 200 ms, confirming the brain’s pre-attentive response to any prosodic modulation. The MMN amplitude was, however, statistically larger to the deviation in affective prosody in comparison to the deviation in pitch accent and affective prosody combined, which is in line with previous research indicating not only a larger MMN response to affective prosody in comparison to neutral prosody but also a smaller MMN response to multidimensional deviants than unidimensional ones. The results, further, showed a significant P3a response to the affective prosody change in comparison to the pitch accent change at around 300 ms, in accordance with previous findings showing an enhanced positive response to emotional stimuli. The present findings provide evidence for distinct neural processing of different prosodic cues, and statistically confirm the intrinsic perceptual and motivational salience of paralinguistic information in spoken communication.

## Introduction

Variations in prosodic features, such as duration, intensity, and fundamental frequency (*f*0), are crucial for spoken communication both at the linguistic and paralinguistic levels. Swedish has for instance two distinctive linguistic prosodic patterns, pitch accents 1 and 2, which are primarily associated with the timing of *f*0, and these accent patterns (indicated with superscripts) occasionally generate lexically distinct minimal pairs as in anden^1^ “the duck” and anden^2^ “the ghost” (Bruce, 2007; Riad, 2014). Paralinguistic information such as vocal affect is also effectively communicated by prosodic modulations; sadness is typically associated with decreased *f*0 and intensity levels, whereas anger is characterized with increased *f*0 and intensity levels (Scherer, 1986; Banse and Scherer, 1996; Juslin and Laukka, 2001, 2003). Given that both linguistic and paralinguistic prosody are rooted in the same acoustic variables, it is crucial to determine how prosodic cues of different origin are extracted and analyzed by the brain. Belyk and Brown (2014), in their statistical meta-analysis of neuroimaging studies, indicated both shared and distinct neural networks involved in the processing of different prosodic functions. The present study adds on previous work by examining neural activity associated with Swedish pitch accents and affective prosody using the electroencephalography (EEG) technique and the mismatch negativity (MMN) and P3a components of event-related potentials (ERPs).

The MMN component is based on an oddball paradigm, where a deviant stimulus is interspersed among frequent standard stimuli, and signals the brain’s automatic reaction to deviations in auditory input at around 100–250 ms after divergence point and with a fronto-central scalp distribution (Näätänen et al., 1978, 2007). The MMN response successfully indicates neural correlates of both low-level acoustic and high-level cognitive processing associated with prosodic information (e.g., Näätänen et al., 1978; Honbolygó et al., 2004, 2020; Weber et al., 2004; Friederici et al., 2007; Zora et al., 2015, 2016a,b, 2019, 2020; Garami et al., 2017). Previous research indicated MMN activation to linguistic prosody change in Swedish words that are distinguished on the sole basis of pitch accent, fasen^1^ “the phase” and fasen^2^ “expletive,” reflecting the activation of different lexical items in the brain based on prosody (Zora et al., 2020). The MMN response was even documented for the relevance of prosody in early morphological processing in Swedish, and specification of stress (lexical vs. phonological) was demonstrated to influence the processing of derivations in the brain (Zora et al., 2019). These MMN results, moving beyond the signal-based perception, pinpoint that linguistic prosody is indeed accommodated in the long-term memory representations (Honbolygó and Csépe, 2013; Zora et al., 2015, 2016a,b, 2019, 2020). Affective prosody was also found to modulate the amplitude of MMN response, being larger for emotional than for neutral vocalizations (Schirmer et al., 2005, 2007; Schirmer and Kotz, 2006; Schirmer and Escoffier, 2010).

The MMN response is often followed by a P300 (P3a) response, with a fronto-central scalp distribution, reflecting attention allocation to unexpected events, as well as salience and contextual novelty of stimuli (Näätänen, 1990; Linden, 2005; Escera and Corral, 2007; Näätänen et al., 2007; Polich, 2007). Changes in linguistic prosody have for instance been shown to be perceptually more salient and therefore, eliciting a larger P3a response as compared to changes in temporal components of speech sounds (Wang et al., 2005). Similarly, a larger P3a response has been indicated to affective prosody as compared to neutral prosody in vowels, pseudowords, and words (Pakarinen et al., 2014; Carminati et al., 2018; Zora et al., 2020). The P3a response has also been argued to reflect not only the perceptual salience of the physical context such as affective prosody, but also all features making the stimulus contextually and motivationally more salient such as emotional semantics (Wambacq and Jerger, 2004). Beyond the P300 response, other positive ERP responses (hereafter called late positive component, LPC) have been reported to emotional stimuli with a time range extending from 300 to 1,000 ms (Cuthbert et al., 2000; Dillon et al., 2006; Fischler and Bradley, 2006; Paulmann et al., 2013; Pell et al., 2015; Steber et al., 2020). Paulmann et al. (2013) indicated that although it can be differentiated as early as 200 ms (see also Paulmann and Kotz, 2008; Paulmann et al., 2010; Schirmer et al., 2013), affective prosody undergoes a detailed analysis to regulate the social interaction, indicated by an LPC response. In our research (Zora et al., 2020), an LPC response was elicited to a match between affective prosody and emotional semantics (swear word uttered with anger in voice), reflecting semantic analysis and memory retrieval rather than simple perceptual salience.

To consolidate and add on these findings, in the present study we examined neural responses to variations in linguistic and affective prosody alone as well as combined using a passive auditory oddball paradigm. A pitch accent contrastive minimal pair in Swedish was used as stimuli. Each lexical item was produced once with a neutral and once with an angry affective prosody. A suppressed cold anger, which is argued to be less intense compared to explosive hot anger (Banse and Scherer, 1996; Hammerschmidt and Jürgens, 2007), was used to minimize the possible effects of inherent acoustic salience on neural responses. In line with the ERP components presented above, distinctive MMN responses are predicted to linguistic and affective prosody, indicating that the human brain discriminates between perceptual attributes of these two distinct functions. In addition, intrinsic perceptual and motivational salience of affective prosody is expected to generate positive ERP responses as P3a and LPC. By investigating the interpretation and integration of linguistic and paralinguistic prosody pre-attentively and in a well-balanced paradigm, the present paper is believed to give a better insight into how the brain codes and processes diverse communicative functions although being rooted in the same acoustic features, and provide a deeper understanding of the symbiotic relationship between functionally different cues during spoken communication.

## Materials and Methods

### Participants

Fifteen female native speakers of Swedish (age range 19–52 years, *M* = 33, *SD* = 9.44) participated in the study. All participants were right hand dominant as determined by the Edinburgh Handedness Inventory (Oldfield, 1971), and self-reported normal development and hearing.

### Ethics Statement

The study followed the ethical guidelines on human subject research, and the experimental protocol was approved by the Stockholm Regional Ethics Committee (2019/05501). Written informed consent was obtained from all the participants before data collection.

### Stimuli

The stimuli consisted of a Swedish word pair, [káttεn]^1^ “the cat” and [káttεn]^2^ “expletive/damn”^1^, which is identical in segmental structure but differs in pitch accent, uttered once with a neutral and once with an angry affective prosody. To eliminate the impact of physical properties of the deviants on neural responses, and to enable the differentiation of obligatory ERP responses from the genuine responses of interest, acoustically identical pseudowords *[táttɛm] and *[táttɛm] were used as controls. A 60-year-old female Swedish speech−language pathologist from Stockholm pronounced all the stimuli. Recordings were conducted in an anechoic chamber using a Brüel & Kjær 1/2″ Free−field Microphone (Type 4189) and the REAPER digital audio workstation (version 5.93; 44.1 kHz/16). Praat (version 6.0.33) was used for acoustic analysis and manipulations (Boersma and Weenink, 2014). Pseudowords were created out of the word stimuli by replacing the initial and final segments /k/ and /n/ with /t/ and /m/. Segmental boundaries were specified by visual inspection of waveforms and Gaussian window broadband spectrograms (bandwidth = 260 Hz), and critical segments were extracted from the relevant context and spliced at zero-crossings. Co-articulation effects were neutralized by adding or removing pulses. Length of each stimulus was 800 ms (10 ms onset/offset ramps).

### Experimental Paradigm and Procedure

**FIGURE 1:**
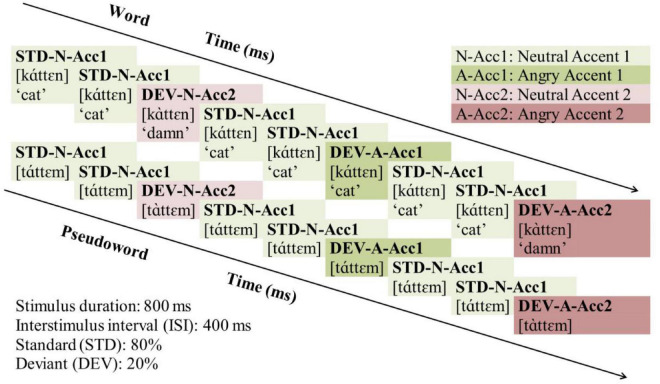
Illustration of the experimental paradigm across Word and Pseudoword blocks. STD, Standard; DEV, Deviant; N, Neutral; Acc, Accent. Neutral stimuli with Accent 1: Light green; Neutral stimuli with Accent 2: Light pink; Angry stimuli with Accent 1: Dark green; Angry stimuli with Accent 2: Dark pink.

The experimental stimuli were presented in a passive auditory oddball paradigm, illustrated across Word and Pseudoword blocks in Figure 1. Standards (STD) were always (N[eutral] stimuli with Acc[ent] 1 (STD-N-Acc1, [káttεn] “the cat”). Deviants (DEV) differed from the standard either only in accent pattern (N[eutral] stimuli with Acc[ent] 2, DEV-N-Acc2, [káttεn] “damn”); or only in anger (A[ngry] stimuli with Acc[ent] 1, DEV-A-Acc1, [káttεn] “the cat”); or both in accent pattern and anger (A[ngry] stimuli with Acc[ent] 2, DEV-A-Acc2, [káttεn] “damn”). The standards formed 80% of the trials (*N* = 1,440) while the deviants 20% (*N* = 360, 120 for each deviant). The deviants were presented pseudo-randomly, with at least two intervening standards between two consecutive deviants. Offset-to-onset interstimulus interval (ISI) was set to 400 ms, and the blocks’ order was counterbalanced across participants.

The EEG paradigm was designed and delivered using the Psychology Software Tool E-Prime (version 2.0). The experimental procedure took place in an electrically shielded and sound-attenuated recording booth. The stimuli were delivered binaurally at a comfortable listening level of 60–65 dB (SPL) through loudspeakers. The task was to ignore the auditory stimuli and focus on a silent documentary (without subtitles) shown on a computer screen. The whole experimental procedure, including breaks and electrode application, took about 2–2.5 h.

### EEG Recordings and Data Analysis

For the EEG data collection, the BioSemi ActiveTwo system and ActiView acquisition software (BioSemi, Netherlands) were employed. Continuous EEG recordings were made from 16 cap-mounted active electrodes (International 10–20 system). A common mode sense active electrode and a driven right leg passive electrode replaced the traditional ground electrode. Four flat-type external electrodes were used for electrooculogram recordings to monitor horizontal and vertical eye movements. In addition, one external electrode was placed on the nose to be used for offline referencing. Offline data analysis was carried out using the EEGLAB toolbox (Delorme and Makeig, 2004) in Matlab (version 9.4) (The Math Works Inc., Natick, Massachusetts, United States). The continuous EEG signal was first filtered (low-pass at 30 Hz and high-pass at 0.5 Hz), and then referenced to the nose electrode. An independent component analysis (Jung et al., 2000) was performed for artifact identification and rejection. The EEG data were then epoched from -100 to 900 ms, relative to the word onset, and a 100 ms pre-onset interval was used for baseline correction. Activation exceeding ± 100μV at any epochs was automatically removed. To plot the ERP waveforms, grand averages were obtained for each stimulus, and deviant-minus-standard subtractions were computed for each deviant.

### Statistical Analysis

The statistical analysis was performed in SPSS (version 24) (International Business Machines Corp., Armonk, New York, United States). Three regions of interest (ROI) were established: Frontal, F3, Fz, and F4; Central, C3, Cz, and C4; and Parietal, P3, Pz, and P4. ERP quantification was computed as a mean voltage within a 50-ms-window centered at peaks in the grand-average waveforms. Time windows were defined to optimally capture ERP modulations related to prosodic changes, and accordingly three consecutive time windows were chosen: 210–260, 300–350, and 570–620 ms. Deviant-minus-standard subtractions were entered in the statistical analysis. To examine whether the MMN responses significantly differed from zero, deviant-minus-standard difference amplitudes were tested against zero with one-sample *t-*tests. A three-way repeated-measures ANOVA with factors of *ROI* (Frontal, Central, and Parietal), *Block* (Word and Pseudoword), and *Deviant* (N-Acc2, A-Acc1, and A-Acc2) was then performed in the three time windows. For significant interactions, follow-up ANOVAs were performed and *post-hoc* pairwise comparisons with Bonferroni corrections were carried out. Greenhouse-Geisser correction was applied in case of sphericity assumption violations. Effect sizes are reported with η^2^ (partial η^2^).

## Results

### Event-Related Potential Data

**FIGURE 2:**
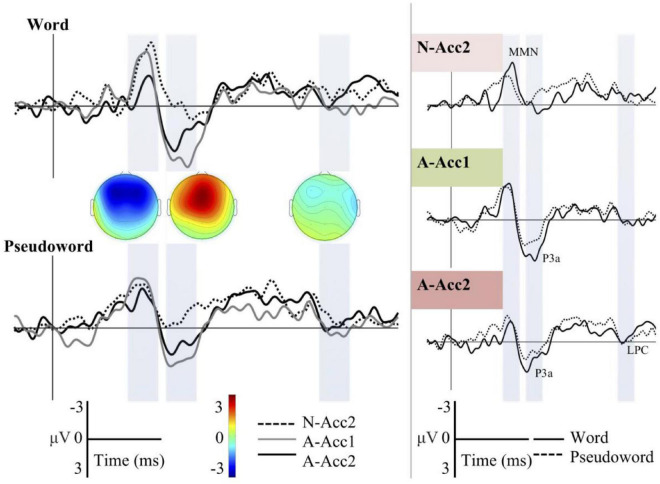
Illustration of the grand average ERP waveforms. Amplitude is given in microvolts [μV, (−3, 3)] and time in milliseconds [ms, (−100, 900)]. Left Panel: Grand average difference waveforms for all the deviants across Word and Pseudoword blocks at the Fz channel. Dotted line: Neutral stimuli with Accent 2 (N-Acc2, i.e., change in linguistic prosody); Gray solid line: Angry stimuli with Accent 1 (A-Acc1, i.e., change in affective prosody); Black solid line: Angry stimuli with Accent 2 (A-Acc2, i.e., change in linguistic–affective prosody). Right Panel: Difference waveforms for both words and pseudowords separately for each deviant (color-coded in accordance with the Figure 1). Black solid line: Word block; Dotted line: Pseudoword block. Shaded bars: Time windows selected for statistical analysis (210–260, 300–350, and 570–620 ms, respectively).

Different waveforms for all deviants across the Word and Pseudoword blocks as well as word-and-pseudoword comparisons for each deviant separately are shown on Figure 2. Grand averages depicted were recorded from Fz. Topographic difference maps are displayed for all three deviants in the Word block to provide a rough estimate of spatial distribution in each time window. Visual analysis of the ERP waveforms indicates that there is a clear negative deflection to all three deviants at around 210–260 ms (1st time window). This response is present in both words and pseudowords and associated with detected changes in the auditory input and therefore called as MMN. However, the variation in response magnitude seen on Figure 2 hints processing differences between the Word and Pseudoword blocks, and the difference appears to be most salient regarding the deviant N-Acc2, i.e., a change in linguistic prosody.

The MMN response is followed by a frontal positive response, prominent especially for the deviants A-Acc1 and A-Acc2, i.e., changes in affective prosody and linguistic-affective prosody, respectively, at around 300–350 ms (2nd time window). This later ERP response, being absent for the neutral affective prosody deviant (N-Acc2), is P3a response, reflecting motivational salience of anger in voice and increased processing as a consequence of this. The comparison of ERPs to words and pseudowords indicates larger response to the word stimuli in both deviants A-Acc1 and A-Acc2. A further slight positive deflection, in line with LPC morphology, seems to be present for the deviant A-Acc2 at around 570–620 ms (3rd time window).

### Statistical Data

**FIGURE 3:**
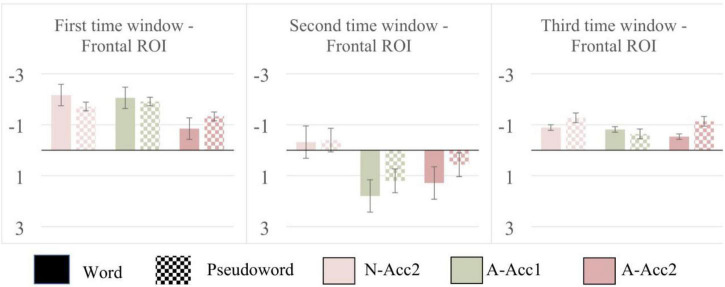
Illustration of the mean and the standard error of the mean for deviant-minus-standard amplitudes (color-coded in accordance with Figures 1, 2) extracted from the frontal electrodes (F3, Fz, F4). Black bars: Word block; Checked bars: Pseudoword block.

Results from repeated-measures ANOVA, follow-up ANOVAs, post-hoc pairwise comparisons and descriptive information are provided as Supplementary Tables 1–3. Mean ERP amplitudes and standard error of the mean are displayed in Figure 3. Results of *t*-tests indicated a significant MMN response for all the deviants in both the Word and Pseudoword blocks: N-Acc2 [*t*_(14)_ = −6.026, *p* < 0.000], A-Acc1 [*t*_(14)_ = −5.701, *p* < 0.000], A-Acc2 [*t*_(14)_ = −2.874, *p* = 0.012] in the Word block, and N-Acc2 [*t*_(14)_ = −4.237, *p* = 0.001], A-Acc1 [*t*_(14)_ = –6.877, *p* < 0.000], A-Acc2 [*t*_(14)_ = −4.206, *p* = 0.001] in the Pseudoword block. Results of ANOVA indicated significant three-way interactions of ROI with Block and Deviant in all the time windows: 1st time window [*F*_(4, 56)_ = 4.265, *p* = 0.027, η^2^ = 0.234]; 2nd time window [*F*_(4, 56)_ = 3.498, *p* = 0.043, η^2^ = 0.200]; and 3rd time window [*F*_(4, 56)_ = 3.376, *p* = 0.049, η^2^ = 0.194]. Follow-up ANOVAs in the 1st time window indicated significant main effects of deviant in the Frontal [*F*_(2, 28)_ = 5.225, *p* = 0.012, η^2^ = 0.272] and Central [*F*_(2, 28)_ = 5.085, *p* = 0.013, η^2^ = 0.266] ROIs. Similarly, in the 2nd time window, significant main effect of deviant was present in the Frontal [*F*_(2, 28)_ = 10.939, *p* < 0.000, η^2^ = 0.439] and Central [*F*_(2, 28)_ = 7.005, *p* = 0.003, η^2^ = 0.334] ROIs. Pairwise comparisons in the Frontal ROI in the 1st time window indicated that the difference between A-Acc1 (*M* = −1.982 μV) and A-Acc2 (*M* = −1.088 μV) was significant (*p* = 0.037). Although the mean amplitude was larger to N-Acc2 (*M* = −1.934 μV) than A-Acc2, the difference between these deviants was not robust enough to reach significance (*p* = 0.078). Comparisons in the central ROI in the 1st time window indicated a significant difference (*p* = 0.005) between A-Acc1 (*M* = −1.974 μV) and A-Acc2 (*M* = −0.971 μV). Pairwise comparisons in the 2nd time window showed a significant difference (*p* < 0.000) between N-Acc2 (*M* = −0.358 μV) and A-Acc1 (*M* = 1.497 μV) and an almost significant difference (*p* = 0.059) between N-Acc2 and A-Acc2 (*M* = 0.925 μV) in the Frontal ROI. Comparison in the Central ROI indicated a significant difference (*p* = 0.002) only between N-Acc2 (*M* = –0.066 μV) and A-Acc1 (*M* = 1.322 μV). Follow-up ANOVA yielded no significant results in the 3rd time window.

## Discussion

The present paper investigated the perception of prosodic modulations of linguistic and affective origin as shown by differences in ERP changes. The overarching aim was to shed light on the neural correlates of pitch accent and angry voice at the early stages of auditory perception, and as such to consolidate and elaborate on earlier work. The results indicated the brain’s automatic reaction to prosodic variations regardless of the origin and wordness. The intrinsic perceptual salience of affective prosody, and enhanced processing of stimuli that carry potentially motivational information was successfully documented. Below are the findings, discussed succinctly on the grounds of previous findings.

The results in the first time window indicated a significant main effect for prosodic deviations, all of which showed a clear negative deflection in the fronto-central brain regions. These changes found are in agreement with the main characteristics of the MMN response; negative going wave of fronto-central maximum, typically peaking at 100–250 ms synchronized to the acoustic change onset (Näätänen et al., 2007). This MMN response, verified also by the *t* tests, confirms the brain’s automatic reaction to prosodic modulations of different origin (be it low level sensory and high level cognitive) in the auditory input, which is in line with previous research (e.g., Näätänen et al., 1978; Honbolygó et al., 2004, 2020; Weber et al., 2004; Friederici et al., 2007; Zora et al., 2015, 2016a,^2020^; Garami et al., 2017).

The pairwise comparisons, however, revealed MMN differences across different prosodic modulations as they indicated a statistically larger MMN response to affective prosody alone as compared to linguistic-affective prosody combination. Although showing a trend only, even linguistic prosody elicited a larger MMN response than linguistic-affective prosody combination. Previous research indicated that multidimensional deviants (intensity and frequency combined) were processed differently and elicited a smaller MMN than unidimensional deviants (intensity and frequency alone) (e.g., Althen et al., 2016). This pattern of results was explained through distinct neural populations participating in the processing of different prosodic cues at the early stages of auditory processing, and then interacting with each other at the later processing stages in the frontal brain areas. Although one might argue that this pattern is in conflict with previous research, indicating additivity of MMN responses to single deviants (e.g., Wolff and Schröger, 2001), it is indeed supported by previous accounts, indexing non/under-additivity for the frontal subcomponent of MMN (Wolff and Schröger, 2001; see also Paavilainen et al., 2003). It has been claimed that the presence of overlapping and interacting brain processes may confound the estimation of MMN response (Paavilainen et al., 2001), and accordingly, we believe that the combination of deviation in different functional levels, namely linguistic and paralinguistic, evoked a complex interaction of various brain processes, which in turn resulted in a decrease in the MMN amplitude. In other words, we argue that a smaller MMN response to the combination of linguistic and affective prosody found in our study indicates that although being rooted in the same acoustic variables, linguistic and affective prosody are processed by distinct neural populations and their interaction has been indicated by a decrease in the MMN amplitude over the frontal brain regions. This argument is in line with the results of neuroimaging studies on the perception of linguistic and affective prosody (see Belyk and Brown, 2014), which indicated differentiation of the two prosodic functions in inferior frontal gyrus. The statistically larger MMN response to affective prosody, produced by all the female participants of our study, is also in line with previous research, indicating an enhanced MMN response to emotional vocalizations as compared to neutral vocalization in women (Schirmer et al., 2005, 2007).

The results found in the first ERP time window showed no significant interaction between any of the deviations and the blocks, indicating that linguistic and affective modulations are processed identically across words and pseudowords. This finding is not in line with an enhanced MMN response to linguistic prosody change in real words in comparison to pseudowords as indicated in our previous research (e.g., Zora et al., 2020). The absence of neural differences regarding linguistic prosody change in the present study might rely on the frequency and etymological differences across stimuli. Given that *katten* as an expletive is non-generic (Borin et al., 2012) and is rooted on the animal cat (SAOB^2^, Ulla Stroh-Wollin, 2008), a change in linguistic prosody might not lead to activation of a separate word in the brain (from “the cat” to “damn/expletive”), although the participants heard the acoustic change from [káttεn]^1^ to [káttεn]^2^.

In the second time window, similar to the first one, a significant main effect for deviants was documented. A positive response of fronto-central maximum was elicited by affective prosody alone and also by affective prosody combined with linguistic prosody. This response clearly reflected anger in the voice since it emerges only as a weak morphological curve to linguistic prosody alone condition (see Figure 3). Given that a non-attended, passive oddball paradigm was used, the positive response found is best interpreted as a P3a response, indicating the allocation of stimulus-driven frontal attention (Polich, 2007) to perceptually salient deviants. Moreover, sensitivity of the P3a to affective prosody is in line with previous studies, documenting a larger P3a response to affective prosody than to neutral prosody (Pakarinen et al., 2014; Carminati et al., 2018; Zora et al., 2020). No significant interaction between any of the deviations and the blocks (i.e., word and pseudoword) was observed, indicating that affective prosody change seems to be treated similarly across words and pseudowords, which is in line with our previous research (Zora et al., 2020).

In spite of the fact that no significant interaction between deviations and blocks (word and pseudoword) was found, the occurrence of a P3a elicited by the affective prosody change might still be influenced by wordness, to some degree at least. The grand average waveforms indicated a larger P3a response to the change in affective prosody in the words than in the pseudowords (Figures 2, 3). Given that *katten* as an expletive is probably rooted on the animal cat, this positive response can still be argued to reflect a match between semantics and the affective prosody as documented as an LPC response in our previous research (e.g., Zora et al., 2020). Beyond the P3a response, the LPC component has been argued to reflect enhanced processing of stimuli that potentially carry relevant emotional information (Cuthbert et al., 2000; Paulmann et al., 2013). Despite a weak morphological trend for the affective prosody combined with linguistic prosody in the third time window, there was no significant positive deflection (Figure 3) as previously reported. Given the absence of an LPC response, we argue that prosodic information might be integrated with the emotional semantics already at an early stage, and accordingly a larger early positive response was elicited to the words than to the pseudowords as an equivalent of a late LPC response. This suggestion is in line with the results of a previous research arguing that the P3a response did not reflect only the perceptual salience, but also the impact of all features on the stimulus’ increased motivational saliency (Wambacq and Jerger, 2004).

To conclude, the present paper indicates that the brain distinguishes between linguistic and affective functions of prosody, hinting distinct neural populations that are involved in the processing of these two functions. The intrinsic perceptual and motivational salience of affective prosody, and enhanced processing of stimuli that carry potentially relevant emotional information have successfully been documented. Future research is, however, warranted employing different stimulus-pairs and using different target languages not only to consolidate the previous and current findings but also to further investigate the resources underpinning the linguistic and affective prosody processing. Given that linguistic and affective prosody occur in parallel in spoken utterances, developing a neural network model of concurrent prosody perception is also of crucial importance. The next step should therefore be to establish how prosodic modulations influence affective and linguistic processing *via* cortical and subcortical pathways using a technique with a good spatial resolution such as functional magnetic resonance imaging.

## Data Availability Statement

The raw data supporting the conclusions of this article will be made available by the authors, without undue reservation.

## Ethics Statement

The studies involving human participants were reviewed and approved by the Stockholm Regional Ethics Committee (2019/05501). The patients/participants provided their written informed consent to participate in this study.

## Author Contributions

HZ: experimental work. HZ and VC: drafting the manuscript and final approval of the version to be published. Both authors contributed to the article and approved the submitted version.

## Conflict of Interest

The authors declare that the research was conducted in the absence of any commercial or financial relationships that could be construed as a potential conflict of interest.

## Publisher’s Note

All claims expressed in this article are solely those of the authors and do not necessarily represent those of their affiliated organizations, or those of the publisher, the editors and the reviewers. Any product that may be evaluated in this article, or claim that may be made by its manufacturer, is not guaranteed or endorsed by the publisher.
