# Supplementary material for: Perception of Prosodic Modulations of Linguistic and Paralinguistic Origin: Evidence From Early Auditory Event-Related Potentials
Source: Front Neurosci. 2021 Dec 23;15:797487. doi: 10.3389/fnins.2021.797487 (PMC8733303; doi:10.3389/fnins.2021.797487)
Supplement: Supplementary file 1 [file Data_Sheet_1.docx]

**Supplementary material**

**Tables**

Table 1 Results for three‐way repeated‐measures ANOVA. Factors consist of region of interest (ROI: Frontal, Central, and Parietal), Block (Word and Pseudoword), and Deviant (Neutral stimuli with Accent 2, N-Acc2; Angry stimuli with Accent 1, A-Acc1; Angry stimuli with Accent 2, A-Acc2) in each time window (210–260 ms, 300–350 ms, and 570–620 ms). Effect sizes are reported with η2 (partial η2). * p < .05

| Window | Factor | *F* | *p* | η^2^ |
| --- | --- | --- | --- | --- |
| 210–260 ms | ROI | *F*(2, 28)=9.557 | 0.005* | 0.406 |
|  | Block | *F*(1, 14)=0.239 | 0.632 | 0.017 |
|  | Deviant | *F*(2, 28)=3.926 | 0.031* | 0.219 |
|  | ROI X Block | *F*(2, 28)=2.180 | 0.155 | 0.135 |
|  | ROI X Deviant | *F*(4, 56)=3.042 | 0.051 | 0.179 |
|  | Block X Deviant | *F*(2, 28)=0.496 | 0.614 | 0.034 |
|  | ROI X Block X Deviant | *F*(4, 56)=4.265 | 0.027* | 0.234 |
| 300–350 ms | ROI | *F*(2, 28)=5.574 | 0.031* | 0.285 |
|  | Block | *F*(1, 14)=2.839 | 0.114 | 0.169 |
|  | Deviant | *F*(2, 28)=6.506 | 0.005* | 0.317 |
|  | ROI X Block | *F*(2, 28)=2.731 | 0.113 | 0.163 |
|  | ROI X Deviant | *F*(4, 56)=7.795 | 0.002* | 0.358 |
|  | Block X Deviant | *F*(2, 28)=1.080 | 0.353 | 0.072 |
|  | ROI X Block X Deviant | *F*(4, 56)=3.498 | 0.043* | 0.200 |
| 570–620 ms | ROI | *F*(2, 28)=21.591 | 0.000* | 0.607 |
|  | Block | *F*(1, 14)=0.516 | 0.484 | 0.036 |
|  | Deviant | *F*(2, 28)=0.998 | 0.381 | 0.067 |
|  | ROI X Block | *F*(2, 28)=0.822 | 0.400 | 0.055 |
|  | ROI X Deviant | *F*(4, 56)=0.127 | 0.926 | 0.009 |
|  | Block X Deviant | *F*(2, 28)=0.042 | 0.959 | 0.003 |
|  | ROI X Block X Deviant | *F*(4, 56)=3.376 | 0.049* | 0.194 |

Table 2 Results for follow-up ANOVAs. Interactions of block (Word and Pseudoword), and Deviant (Neutral stimuli with Accent 2, N-Acc2; Angry stimuli with Accent 1, A-Acc1; Angry stimuli with Accent 2, A-Acc2) were reported in all the three time windows (210–260 ms, 300–350 ms, and 570–620 ms) in each region of interest (ROI, Frontal, Central, and Parietal). Effect sizes are given with η2 (partial η2). * p < .05

| Window | ROI | Factor | *F* | *p* | η^2^ |
| --- | --- | --- | --- | --- | --- |
| 210–260 ms | Frontal | Block | *F*(1, 14)=0.030 | 0.866 | 0.002 |
|  |  | Deviant | *F*(2, 28)=5.225 | 0.012* | 0.272 |
|  |  | Block X Deviant | *F*(2, 28)=1.764 | 0.190 | 0.112 |
|  | Central | Block | *F*(1, 14)=0.052 | 0.823 | 0.004 |
|  |  | Deviant | *F*(2, 28)=5.085 | 0.013* | 0.266 |
|  |  | Block X Deviant | *F*(2, 28)=0.511 | 0.605 | 0.035 |
|  | Parietal | Block | *F*(1, 14)=2.119 | 0.167 | 0.131 |
|  |  | Deviant | *F*(2, 28)=0.981 | 0.366 | 0.065 |
|  |  | Block X Deviant | *F*(2, 28)=1.126 | 0.324 | 0.074 |
| 300–350 ms | Frontal | Block | *F*(1, 14)=4.129 | 0.062 | 0.228 |
|  |  | Deviant | *F*(2, 28)=10.939 | 0.000* | 0.439 |
|  |  | Block X Deviant | *F*(2, 28)=1.208 | 0.314 | 0.079 |
|  | Central | Block | *F*(1, 14)=3.998 | 0.065 | 0.222 |
|  |  | Deviant | *F*(2, 28)=7.005 | 0.003* | 0.334 |
|  |  | Block X Deviant | *F*(2, 28)=1.793 | 0.185 | 0.114 |
|  | Parietal | Block | *F*(1, 14)=0.317 | 0.582 | 0.022 |
|  |  | Deviant | *F*(2, 28)=1.243 | 0.304 | 0.082 |
|  |  | Block X Deviant | *F*(2, 28)=1.368 | 0.271 | 0.089 |
| 570–620 ms | Frontal | Block | *F*(1, 14)=1.965 | 0.183 | 0.123 |
|  |  | Deviant | *F*(2, 28)=1.756 | 0.191 | 0.111 |
|  |  | Block X Deviant | *F*(2, 28)=1.465 | 0.248 | 0.095 |
|  | Central | Block | *F*(1, 14)=0.448 | 0.514 | 0.031 |
|  |  | Deviant | *F*(2, 28)=0.477 | 0.625 | 0.033 |
|  |  | Block X Deviant | *F*(2, 28)=0.064 | 0.938 | 0.005 |
|  | Parietal | Block | *F*(1, 14)=0.015 | 0.903 | 0.001 |
|  |  | Deviant | *F*(2, 28)=0.781 | 0.468 | 0.053 |
|  |  | Block X Deviant | *F*(2, 28)=1.042 | 0.366 | 0.069 |
|  |  |  |  |  |  |
|  |  |  |  |  |  |
|  |  |  |  |  |  |
|  |  |  |  |  |  |
|  |  |  |  |  |  |
|  |  |  |  |  |  |
|  |  |  |  |  |  |

Table 3 Results for pairwise comparisons. The significant main effects of the Deviant in the Frontal and Central ROIs in the first and second time windows (Table 2) were further investigated through pairwise comparisons. Mean values (M) are reported with standard deviations (SD). * p < .05

| Time window | ROI | Comparison | *p* | Level | *M* | *SD* |
| --- | --- | --- | --- | --- | --- | --- |
| 210–260 ms | Frontal | N-Acc2 – A-Acc1 | 1.000 | N-Acc2 | –1.934 | 0.304 |
|  |  | N-Acc2 – A-Acc2 | 0.078 | A-Acc1 | –1.982 | 0.266 |
|  |  | A-Acc1 – A-Acc2 | 0.037* | A-Acc2 | –1.088 | 0.276 |
|  | Central | N-Acc2 – A-Acc1 | 0.471 | N-Acc2 | –1.487 | 0.300 |
|  |  | N-Acc2 – A-Acc2 | 0.493 | A-Acc1 | –1.974 | 0.233 |
|  |  | A-Acc1 – A-Acc2 | 0.005* | A-Acc2 | –0.971 | 0.254 |
| 300–350 ms | Frontal | N-Acc2 – A-Acc1 | 0.000* | N-Acc2 | –0.358 | 0.273 |
|  |  | N-Acc2 – A-Acc2 | 0.059 | A-Acc1 | 1.497 | 0.301 |
|  |  | A-Acc1 – A-Acc2 | 0.613 | A-Acc2 | 0.925 | 0.503 |
|  | Central | N-Acc2 – A-Acc1 | 0.002* | N-Acc2 | –0.066 | 0.183 |
|  |  | N-Acc2 – A-Acc2 | 0.418 | A-Acc1 | 1.322 | 0.268 |
|  |  | A-Acc1 – A-Acc2 | 0.212 | A-Acc2 | 0.598 | 0.402 |
